# Impact of structured reporting on developing head and neck ultrasound skills

**DOI:** 10.1186/s12909-019-1538-6

**Published:** 2019-04-11

**Authors:** Benjamin P. Ernst, Fabian Katzer, Julian Künzel, Mohamed Hodeib, Sebastian Strieth, Jonas Eckrich, Anna Tattermusch, Matthias F. Froelich, Christoph Matthias, Wieland H. Sommer, Sven Becker

**Affiliations:** 1 Department of Otorhinolaryngology, University Medical Center Mainz, Langenbeckstraße 1, 55131 Mainz, Germany; 2 Department of Radiology, LMU University Hospital, Marchioninistraße 15, 81377 Munich, Germany; 3 Smart Reporting GmbH, Brienner Straße 13, 80333 Munich, Germany; 4 Institute of Clinical Radiology and Nuclear Medicine, Institute of Clinical Radiology and Nuclear Medicine, Faculty Mannheim-Heidelberg University, Theodor-Kutzer-Ufer 1-3, 68167, Mannheim, Germany; 5 Department of Otolaryngology, Head and Neck Surgery, University of Tübingen, Elfriede-Aulhorn-Straße 5, 72076 Tübingen, Germany

**Keywords:** Medical education, Ultrasonography, Head and neck Cancer, Salivary gland diseases, Lymphadenopathy

## Abstract

**Background:**

Reports of head and neck ultrasound examinations are frequently written by hand as free texts. This is a serious obstacle to the learning process of the modality due to a missing report structure and terminology. Therefore, there is a great inter-observer variability in overall report quality. Aim of the present study was to evaluate the impact of structured reporting on the learning process as indicated by the overall report quality of head and neck ultrasound examinations within medical school education.

**Methods:**

Following an immersion course on head and neck ultrasound, previously documented images of three common pathologies were handed out to 58 medical students who asked to create both standard free text reports (FTR) and structured reports (SR). A template for structured reporting of head and neck ultrasound examinations was created using a web-based approach. FTRs and SRs were evaluated with regard to overall quality, completeness, required time to completion and readability by two independent raters (Paired Wilcoxon test, 95% CI). Ratings were assessed for inter-rater reliability (Fleiss’ kappa). Additionally, a questionnaire was utilized to evaluate user satisfaction.

**Results:**

SRs received significantly better ratings in terms of report completeness (97.7% vs. 53.5%, *p* < 0.001) regarding all items. In addition, pathologies were described in more detail using SRs (70% vs. 51.1%, *p* < 0.001). Readability was significantly higher in all SRs when compared to FTRs (100% vs. 54.4%, *p* < 0.001). Mean time to complete was significantly lower (79.6 vs. 205.4 s, *p* < 0.001) and user satisfaction was significantly higher when using SRs (8.5 vs. 4.1, *p* < 0.001). Also, inter-rater reliability was very high (Fleiss’ kappa 0.93).

**Conclusions:**

SRs of head and neck ultrasound examinations provide more detailed information with a better readability in a time-saving manner within medical education. Also, medical students may benefit from SRs in their learning process due to the structured approach and standardized terminology.

## Background

The concept of structured reporting has been advocated for various diagnostic modalities over the past decade [1–5]. According to generally accepted definitions, a structured report (SR) consists of, inter alia, standardized headings, sub-categories to specify results and, most importantly, a standardized language [6, 7]. There is a great demand for innovative reporting strategies to compensate the current lack of training in reporting [8, 9]. Numerous studies have pointed out the superiority of SRs in terms of report completeness, accuracy and time-efficiency when compared to hand-written free text reports (FTR) [2, 10–12]. The underlying templates for structured reporting contain standardized chapters and terminology. This reduces the likelihood of missing key structures during the examination as well as poor descriptions during report generation [13, 14]. Consequently, SRs have a great potential for diagnostic modalities that follow a standardized workflow. This includes head and neck ultrasound examinations, the gold standard for routine outpatient diagnostics of various pathologies [15–20]. Due to frequent use within follow-ups, precise and comparable reports are of central importance [21]. Consequently, structured reporting may be of great benefit, especially during the learning process, by offering a standardized approach to both the examination and report generation [2, 21]. The standardized structure also makes SRs eligible for scientific big data analyses [13]. Frequently, inexperienced examiners benefit of using SRs which leads to more complete reports [21, 22]. This is supported by multiple studies showing a preference for SRs by both the examining and referring physician due to a higher degree of accuracy and comprehensiveness of the pathology [21, 23–25].

Head and neck ultrasound marks a very complex examination technique. The extent of clinically relevant structures as well as the recommended terminology may be unclear to the inexperienced examiner [2, 18]. Therefore, the use of SRs may be of help over the course of the learning process. There is evidence, that structured reporting reduces the number of missed pathologies [13, 26, 27].

A frequently criticized aspect of structured reporting is that it may be too rigid and adaptations may turn out unprecise and also not time-efficient [6, 26]. This is emphasized by the complexity of the examination, the high level of work routine and the great number of structures that have to be examined for various disorders such as head and neck cancer, carotid artery stenosis and thyroid diseases [15, 16, 20].

It is yet unknown whether structured reporting should be implemented at any certain level of training (i.e. medical school, residency etc.) or whether an early implementation is associated with a steeper learning curve. In consequence, the aim of the present study was to evaluate the impact of SRs on the learning process. We followed the hypothesis that a learning process is defined by acquiring new knowledge and skills that ultimately influence attitudes, decisions and actions [28]. In this context, change in overall quality, completeness of content, time required for completing the report and readability of head and neck ultrasound examinations were assumed to be indicators of the modality’s learning process. Additionally, we evaluated the medical students’ satisfaction of using either SRs or FTRs.

## Methods

### Study design

The present study compared FTRs of head and neck ultrasound examinations to SRs within a medical school educational concept. The University Medical Center Mainz hosts various annual immersion courses on ultrasound diagnostics for medical students with a pronounced interest in the modality. In total, 58 medical students participated in our annual 2018 immersion course on head and neck ultrasound (see Table 1) who all agreed to take part in this study. The course included extensive training in both conducting and reporting head and neck ultrasound examinations. The level of experience regarding ultrasound was evaluated in the beginning of the course by self-assessment using a five-point scale (5: very high experience, 0: insufficient experience). Medical students were trained to report using FTRs which represents our department’s standard. Participants were randomly assigned to documented images of three different common head and neck pathologies. The images were obtained ahead of our annual immersion course during routine outpatient care at our department.

**Table 1:** Demographics and characteristics of participating medical students

Characteristics	Value
Number of participating students	58
Years since enrolment in medical school (mean ± SD)	2.71 ± 0.81 years
	(range: 1–4 years)
Gender	Male: 44.8%, Female: 55.2%
Self-assessment of experience in ultrasound	Insufficient: 0%
	Poor: 17.2%
	Moderate: 58.6%
	High: 24.2%
	Very High: 0%

These pathologies included an unspecific cervical lymphadenitis, a benign tumor of the parotid gland as well as a solitary submandibular duct calculus. In a first step, FTRs (*n* = 58) of the assigned pathologies were created analogously to the training within the immersion course. In a second step, participants used the same images to generate corresponding SRs (*n* = 58). This sequence was chosen in order to reduce bias since, unlike structured reporting, free text reporting does not offer any feedback to the user. Participating students completed a user satisfaction questionnaire immediately after finalizing FTRs and SRs.

### Sample size calculation

As previously described in the literature, the number of participants needed was calculated based on the anticipated effect size when comparing the percentage of FTRs with 80% completeness or higher to SRs [29]. We assumed that 55% of FTRs would have very high completeness ratings (i.e. of 80% or higher), considering the report quality of other imaging techniques as published in the literature [21, 29]. Additionally, we estimated that the ratio of very high completeness ratings would go up to 70% using SRs. The power was set to 80% with a significance level of α = 0.05. Using these parameters, the minimum number of reports required in the study was calculated to be *n* = 82 (41 reports in each group) [30].

### Image acquisition

Images of common head and neck pathologies were previously acquired in our outpatient department using a LOQIQ E9 ultrasound unit (GE Healthcare, Little Chalfont, United Kingdom) with a 9 MHz linear transducer. Images were stored and reviewed using a web-based picture archiving and communication system (PACS, Sectra AB, Linköping, Sweden).

### FTRs and SRs

Our department’s standard template was used which is to be completed in writing to create FTRs. For SRs a web-based software (Smart Reporting GmbH, Munich, Germany, http://www.smart-radiology.com) was used to create a specific template for structured reporting of head and neck ultrasound examinations. The template was designed by three board-certified otorhinolaryngologists with a high expertise in ultrasound examinations. It was based on the most recent recommendations of the German Society for Ultrasound in Medicine for reported structures and terminology. The template was created to address a wide variety of pathologies. The user is guided through a clickable decision tree specifically designed for the diagnostic modality. Therefore, structures and pathologies are addressed uniformly in every report.

**Fig. 1:**
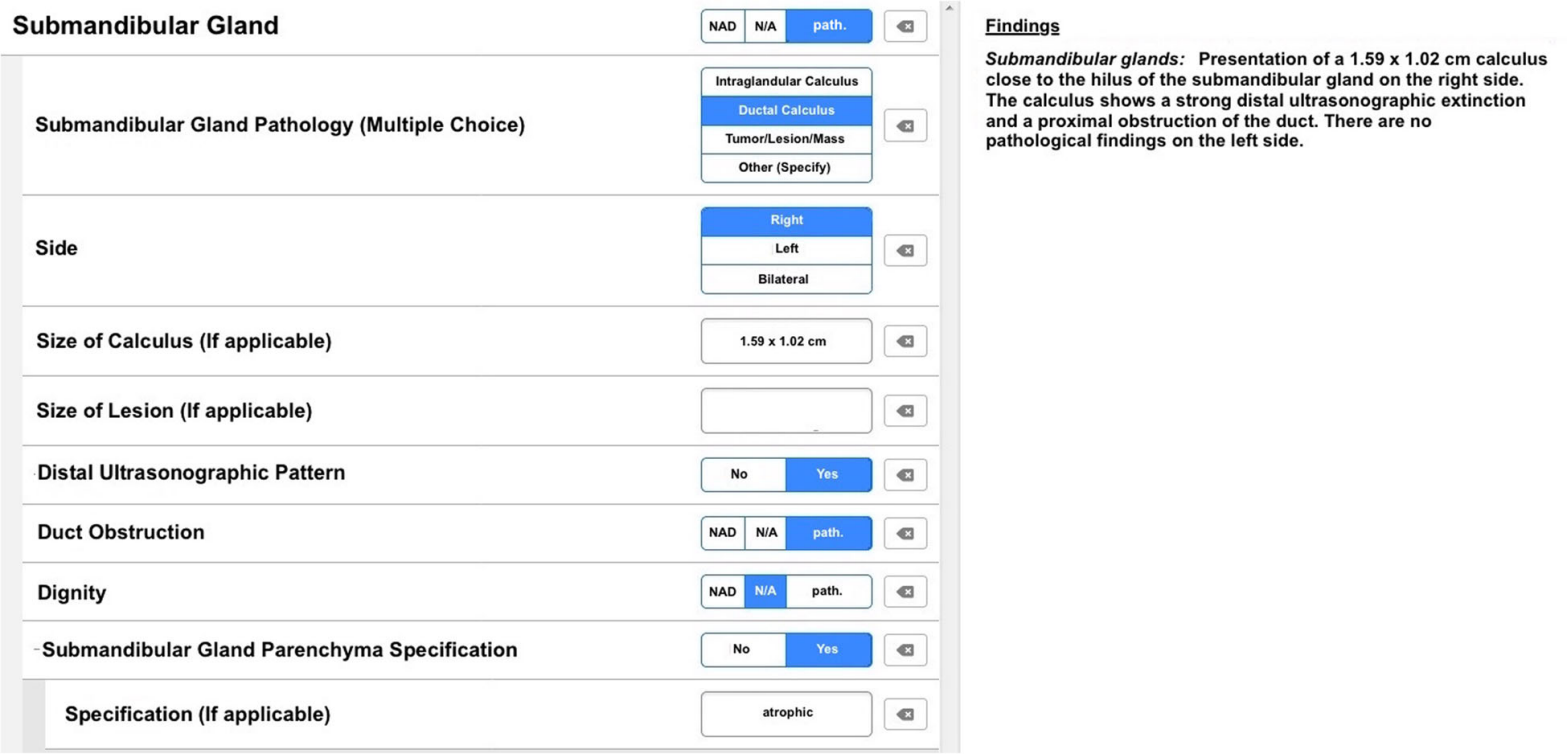
Screenshot of the decision tree within the reporting software. Shown is an exemplary report of submandibular duct pathology. On the left side, the examiner can select the type of pathology, side, size as well as pathological feature such as distal ultrasound pattern, duct obstruction and assessment of dignity, while the template generates full semantic sentences on the right side

By working through the decision tree, the software generates full sentences using previously defined text modules (see Fig. 1). Free text elements may be added to enable a maximum degree of flexibility. Additionally, info boxes provide background information and may be used to show sample pictures or clinical guidelines. This feature makes it less likely to consult colleagues or further medical literature during the report [31].

### Report evaluation

Time required to complete the report was documented in the course of reporting. The 116 anonymized reports (58 FTRs and SRs each) were evaluated for overall completeness (i.e. reporting of bilateral neck levels, salivary glands and major blood vessels), level of pathological detail and readability by two independent board-certified otorhinolaryngologists using a standardized evaluation template. The template was created by three highly experienced sonographic examiners. Overall report quality was defined as the blend of overall report completeness, level of detail and readability and categorized as insufficient (0–20% overall report quality), poor (20–40%), moderate (40–60%), high (60–80%) or very high (80–100%). Readability was subjectively assessed using a five-point scale (5: very high readability, 0: insufficient readability).

Besides, we implemented a user satisfaction questionnaire using a ten-point visual analogue scale (VAS, 10: Complete agreement, 0: Complete disagreement). Participating medical students were asked about practicability (question 1), usefulness in everyday practice (question 2), improvement in report-quality (question 3), time-effectiveness (question 4), justification of additional time needed (if applicable, question 5), benefits for inexperienced physicians conducting (question 6) and reporting (question 7) ultrasound examinations of the head and neck, usability by intuition (question 8) and clarity of arrangement of the template (question 9).

### Statistical analysis

Data are reported as the mean percentage of maximum outcome (i.e. percentage of maximum quality, completeness and detail), mean time required to report (seconds) and mean VAS values ± SD. Wilcoxon signed-rank test for paired nominal data was used to compare overall completeness, level of detail, time required as well as VAS scores of questionnaires. Linear regression analysis was applied to determine correlations. A *p*-value of less than 0.05 was considered statistically significant. Fleiss’ kappa was used to evaluate inter-rater reliability [32, 33]. All statistical analyses were performed using SigmaPlot 12 (Systat Software, Inc., San Jose, CA, USA).

## Results

Overall 116 reports (*n* = 58 for SRs and FTRs each) were eligible for analysis. All reports were assessed by two board-certified otorhinolaryngologists resulting in a total of *n* = 332 ratings (*n* = 116 ratings per reviewer).

### Report analysis

Report analysis showed that using SRs results in a significantly higher completeness in all categories (97.7% vs. 53.5%, *p* < 0.001). In detail, SRs showed higher completeness in terms of lymph nodes (95.1% vs. 33.5%, *p* < 0.001), salivary glands (99.7% vs. 83.3%, *p* = 0.002) and major blood vessels (100% vs. 61.2%, *p* < 0.001). Also, pathologies were described in significantly greater detail (70% vs. 51.1%, *p* < 0.001) and mean time required for reporting was significantly shorter when using SRs (79.6 s vs. 205.4 s, *p* < 0.01). SRs were rated to have a significantly higher readability (100% vs. 54.4%, *p* < 0.001) when compared to FTRs.

**Fig. 2:**
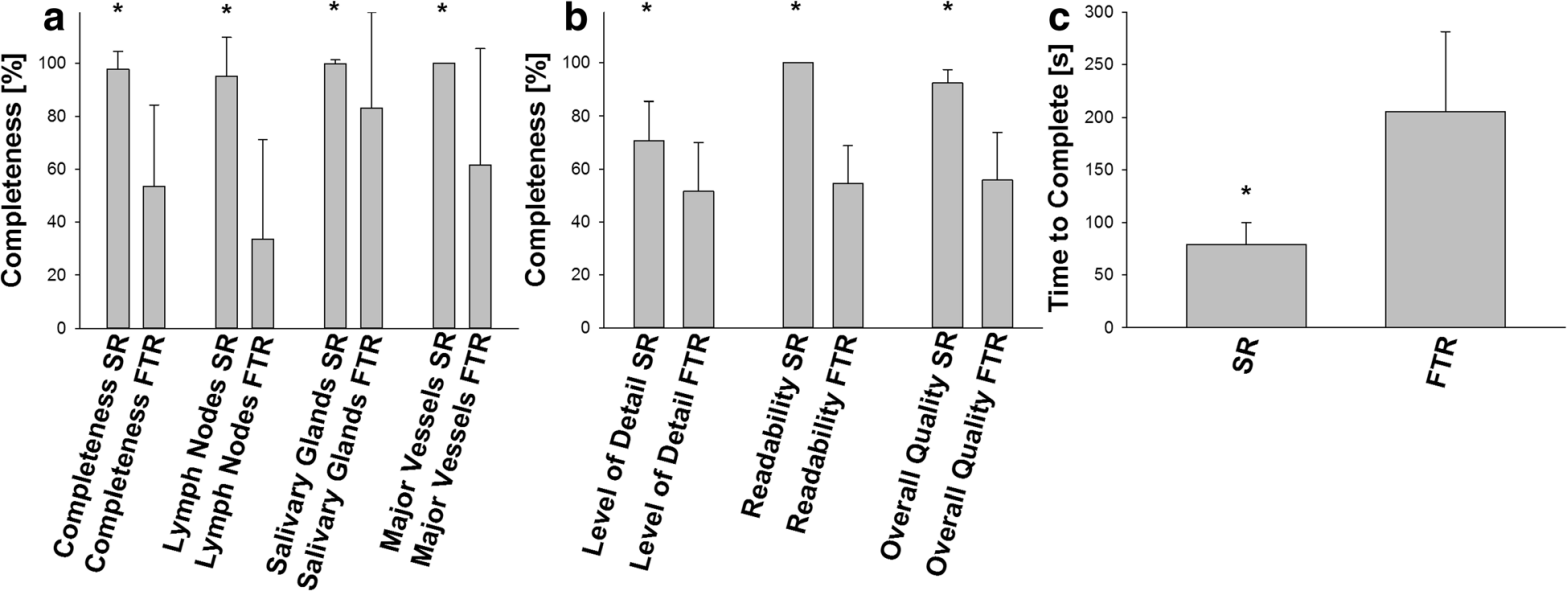
Results of report analysis. Structured reports (SR) yield significantly higher completeness ratings in describing cervical lymph nodes, major neck vessels and salivary glands resulting in a significantly increased overall completeness (**a**). Moreover, level of pathological detail, readability and overall report quality was significantly higher when using SRs (**b**). Time needed to complete the report was also significantly shorter when using SRs (**c**). * *p* < 0.05

Subsequently, overall report quality was determined and reports were categorized as described above. Using SRs resulted in a significantly increased mean overall report quality when compared to FTRs (92.3% vs. 55.8%, *p* < 0.001). There was a significant association of poor to moderate report quality with FTRs (48.3% vs. 0%, *p* < 0.001) while high to very high report quality was significantly associated with SRs (100% vs 10.3%, *p* < 0.001). Also, linear regression analysis revealed no significant correlation between time to complete the report and overall report quality (R = 0.193, R^2^ = 0.0371, *p* = 0.317). A detailed report analysis is shown in Fig. 2. Inter-rater reliability was very high with a Fleiss’ kappa of 0.93.

### User satisfaction

**Fig. 3:**
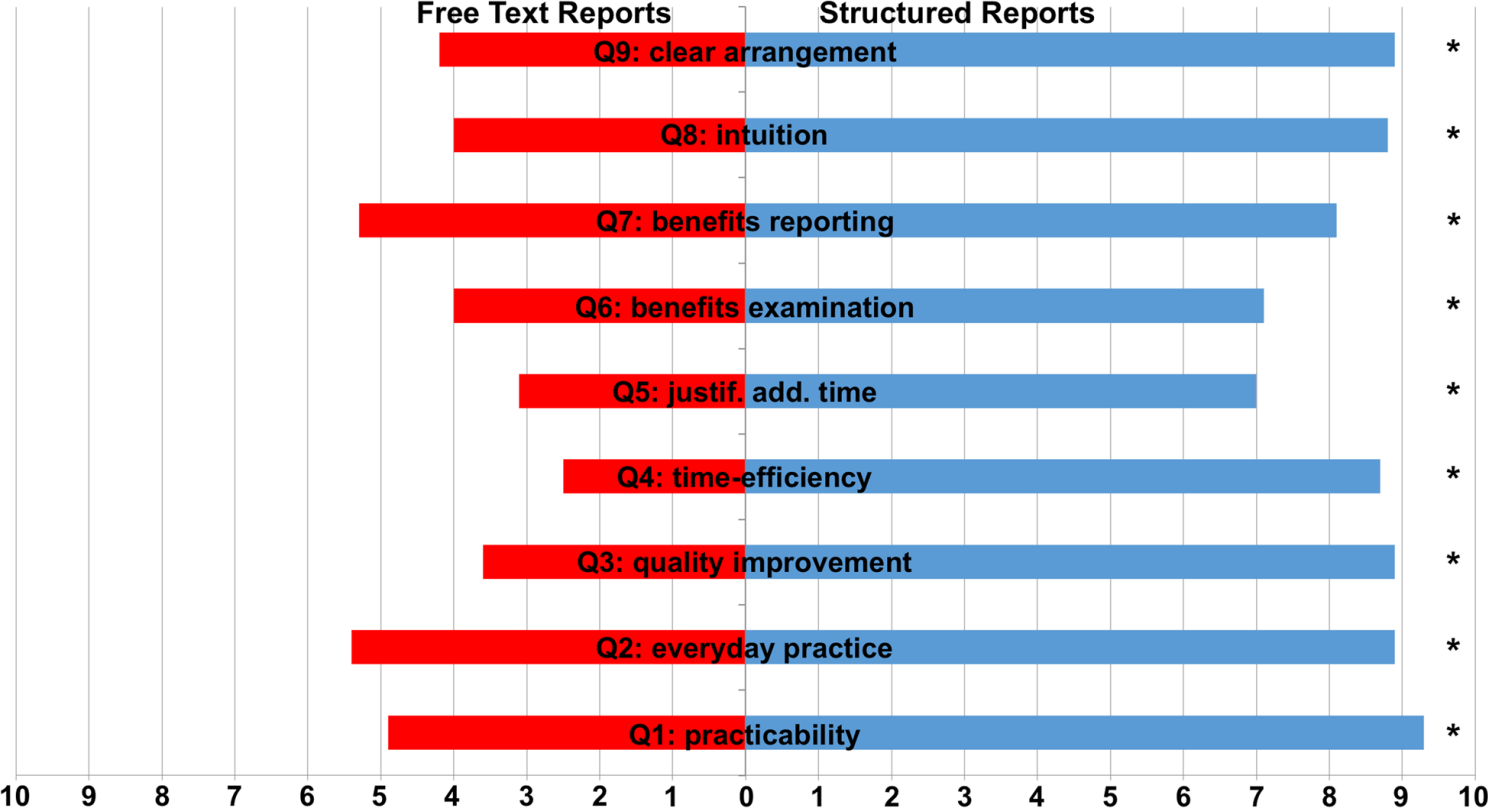
Visual analog scale (VAS) of questionnaire findings. User satisfaction of the 58 participating medical students was evaluated with a questionnaire using a visual analog scale (VAS, 10: Complete agreement, 0: Complete disagreement). Participants were asked about practicability (Q1: practicability), usefulness in everyday practice (Q2: everyday practice), improvement in report-quality (Q3: quality improvement), time-efficiency (Q4: time-efficiency), justification of additional time needed (if applicable, Q5: justif. Add. time), benefits for inexperienced physicians conducting (Q6: benefits conducting) and reporting (Q7: benefits reporting) ultrasound examinations of the head and neck, usability by intuition (Q8: intuition) and clarity of arrangement (Q9: clear arrangement) of structured reports (right side, blue bars) and free text reports (left side, red bars). The questionnaire revealed a significant preference for structured reports in all categories. * *p* < 0.05

The questionnaire revealed significant preference for SRs by all interviewed users in all categories (VAS 8.5 vs. 4.1, *p* < 0.001). The use of SRs was regarded as applicable for everyday use (9.1 vs. 5.1, *p* < 0.001), as time-efficient (7.8 vs. 3.0, *p* < 0.001) and intuitive (8.8 vs. 4.0, *p* < 0.001). Moreover, SRs were considered to be supportive for medical students in both conducting the examination (7.1 vs. 4.0, *p* = 0.003) and generating the report (8.1 vs. 5.3, *p* < 0.001). Consequently, structured reporting was thought to produce reports with a higher level of quality (8.9 vs. 3.6, *p* < 0.001). A detailed analysis of questionnaires is shown in Fig. 3.

## Discussion

Head and neck ultrasound defines the clinical standard for routine outpatient medical imaging in otorhinolaryngology. Its value for the evaluation of various diseases of the neck has been pointed out by multiple studies [15–20]. At best, basic examination skills are taught during medical school. Consequently, there is a lack of teaching the report generation itself resulting in poor report quality [8]. This contrasts the importance of the actual report and its potential implications on clinical decision-making.

Ultrasound examinations of the head and neck depict a highly complex imaging technique. There is a large number of important structures within a rather small space. Their topographic relationship and its importance may not be clear to inexperienced examiners. This lack of knowledge is not limited to the examination. It also includes the report, as well as its structural content and language. Therefore, the use of structured reporting may facilitate the learning process by leading unskilled examiners through the examination and the reporting by revealing relevant content and appropriate terminology [34]. This is advocated by multiple publications showing that less pathologies are overlooked during an exam and correlating SRs with a higher diagnostic accuracy [13, 26, 35]. Due to a rather low intra- and interrater reliability of FTRs, it has been suggested that structured reporting is the key to improve medical reporting significantly [2, 24].

Aim of the study was to evaluate impact of structured reporting of head and neck ultrasound examinations on the learning process during medical school education. A dedicated focus was set on overall quality, completeness, detail, readability as well as time-efficiency and user satisfaction. To the best of our knowledge, there have been no previous studies on the impact of structured reporting on the learning process.

Our data showed that the use of SRs results in a significantly improved overall report quality, completeness and readability. Furthermore, medical students were able to describe pathologies in significantly greater detail while using the recommended terminology. Mean time to complete the report was also significantly reduced by using SRs. Analysis of user satisfaction revealed a clear preference for SRs.

These results are in line with previous studies that were able to demonstrate a correlation between structured reporting and high report quality in various diagnostic modalities [21, 23–25, 29, 36]. Our results also show that inexperienced examiners highly prefer SRs. Possible reasons for that may include the standardized appearance, language and output as well as the implementation of clinical principles and guidelines.

An important topic of discussion is whether SRs may prove to be too rigid within clinical application where a high degree of flexibility is needed [22]. Furthermore, linguistic quality may be impaired by semi-automatic generation of semantic sentences based on decision trees. Concerns are backed by numerous publications that reported non-inferior report quality and superior linguistic quality for FTRs [6, 11, 26]. The latter may be overcome by the precise planning of decision trees and the use of recommended terminology as well as advanced information technology [2]. These factors are key to achieve high quality reports using adequate language. Appropriate information technologies may incorporate crosslinking possibilities and free text elements to ensure maximum completeness, time-wise efficiency and degree of flexibility. Terminology and phrasing should be discussed between examining and referring physicians ahead of implementation. This ensures a high level of user satisfaction and comprehension of reports as outlined by our results. It also results in reports with virtually no grammatical or orthographical mistakes. This may be beneficial for inexperienced young residents and to non-native speakers.

The problem of non-native speaking examiners is emphasized by the increasing importance of telemedical consulting [37]. Teleradiological reporting has become a necessity for rural areas with a shortage of specialists. This problem may be overcome by broadband connections that enable the transfer of huge amounts of data that may be interpreted and reported in other regions, whether domestic or foreign. In the case of foreign countries, reporting specialists may not have adequate linguistic skills to create high quality reports or to answer queries of referring physicians. In consequence, SRs may be a key factor in overcoming poor report quality due to limited language skills [38, 39].

There is some evidence that the rather rigid reporting conditions of SRs may be of benefit during the learning process [34]. Hence, our results indicate a potential positive influence of SRs on the learning process. Medical students were able to create more complete reports in significantly shorter time frames. Whether these findings imply that using structured reporting leads to more thorough examinations needs to be subject of future research.

Furthermore, SRs have a favorable time-efficiency. Possible explanations for the increased time-efficiency may include the pre-defined structure. The clickable decision tree is redundant for every report and facilitates a better workflow. Additionally, the use of structured reporting prevents inexperienced examiners from wasting time on the structure, content and terminology of the report. The time-saving aspect of structured reporting is in line with numerous publications, especially for unremarkable findings or common pathologies [25, 40]. On the other hand, there is evidence that SRs may be more time consuming in complex cases due to pathological features that are not addressed in the template and have to be reported by using free text elements [41]. These concerns do not apply entirely within the training process of the modality. Inexperienced examiners who are not provided with a structure and correct terminology may get lost while describing pathologies they are unfamiliar with. This may very well lead to either a very long time required to complete the report, the consultation of other physicians at hand or a low report quality because features of the disease are not addressed. All of these concerns represent common causes of workflow impairment. A decrease in frustration for the examiner may also play a role in the significant preference for SRs in our results.

Additionally, our data are discordant with the hypothesis of other studies that implementation of SRs results in an increased time required for reporting [42]. Most studies involving structured reporting are carried out by physicians familiar with FTRs for years. This results in a bias because of the overall faster reporting using FTRs [42]. Therefore, the change in workflow has to be taken into consideration since it is known to cause a significant initial loss of time due to the learning effects of the new modality [22]. This initial loss is known to be put in perspective within a certain timeframe and often results in a more efficient workflow after adaptation is completed [42]. Additionally, disciplines with large numbers of referred examinations, like radiology, pathology or internal medicine, struggle with queries due to incomplete or misinterpreted reports. It has been demonstrated that SRs are widely time-efficient after the initial setback and that implementation eventually leads to significantly faster reporting and fewer queries [42]. Since our study evaluated medical students unfamiliar with both SRs or FTRs the potential bias of being used to either one of them can be ruled out. Consequentially, our data showed a faster time to complete using structured reporting right from the start without the previously described initial setback. Therefore, the previously described initial loss of time cannot be exclusively attributed to the use of SRs but rather to the fact that most physicians have been trained to FTRs over the past decades [42].

At last, the participating medical students unanimously stated that structured reporting seems to cause an increase in report quality and time-efficiency. The hypothesis that a higher level of report quality may lead to an improved outcome for the patient has to be answered by future studies. This hypothesis is supported by the fact that structured reporting has been shown to promote the use of clinical guidelines, thus, endorsing evidence-based medicine [22, 34].

### Limitations

Since this study investigated a single cohort of medical students, certain limitations apply. The cohort consisted of German students of different years in medical school, so a homogeneous medical knowledge as well as ultrasound skills cannot be assumed. In addition, the course was not perfectly balanced gender-wise. The annual extra-curricular immersion course on head and neck ultrasound at the University Medical Center Mainz is typically taken by students with a pronounced interest both in otorhinolaryngology and especially in ultrasound. This marks a potential bias because the cohort may not reflect an average cohort of medical students. Additionally, medical students were provided with standardized images of common head and neck pathologies and did not perform the ultrasound examination and image acquisition themselves. Ultrasound represents a highly dynamic imaging technique which is bound to be dependent on the examiner. Therefore, reporting on standardized images is not entirely representative, since different examiners will document and therefore report on different images. Since participating students used the same images for SRs and FTRs, potential bias due to testing or learning effects cannot be ruled out. Therefore, the sequence of reporting within this study was chosen to minimize these effects because of the absence of feedback from FTRs. Furthermore, completeness of FTRs relies heavily on the knowledge of the examiner, since correct content and language have to be obeyed. This does not apply completely to SRs, since these details are implemented in the software. Our annual immersion course teaches basic and advanced knowledge in this field but it may not compensate for pre-existing differences in expertise or level of attention during the course. Consequently, results of FTRs may be underrepresented.

## Conclusion

In conclusion structured reporting seems to be a promising approach to generate high-quality, detailed and comparable reports, especially in the context of medical education. The reduced time needed to complete the report reflects the intuitive use of the template used in the present study and may lead to a more efficient workflow. This is also supported by the significant preference for SRs by medical students and the general belief that structured reporting enhances the learning process of both the examination and report generation. Consequently, we recommend the implementation of SRs of head and neck ultrasound examinations as the standard for report generation in clinical practice as well as in medical education.

## Abbreviations

ACE: External carotid artery
ACI: Internal carotid artery
FTR: Free text report
GPA: Parotid gland
GSM: Submandibular gland
SR: Structured report
VAS: Visual analog scale
